# Unterschiedliche Erwartungen an die präklinische Versorgung verletzter Kinder? Ein Vergleich zwischen Kinderchirurgen und Unfallchirurgen

**DOI:** 10.1007/s00104-025-02352-6

**Published:** 2025-07-11

**Authors:** Ralf Kraus, Melanie Markmann, Jens Günter Riedel, Emmanuel Schneck

**Affiliations:** 1 Unfall‑, Hand- und Wiederherstellungschirurgie, Universitätsklinikum Gießen und Marburg GmbH, Standort Gießen, Rudolf Buchheim Str. 7, 35385 Gießen, Deutschland; 2 Klinik für Anästhesiologie und operative Intensivmedizin, Universitätsklinikum Gießen und Marburg GmbH, Standort Gießen, Gießen, Deutschland; 3 Klinik für Kinderchirurgie, Universitätsklinikum Gießen und Marburg GmbH, Standort Gießen, Gießen, Deutschland

**Keywords:** Kindertraumatologie, Rettungsdienstliche Versorgung, Ruhigstellung, Schmerztherapie, Reposition, Pediatric traumatology, Rescue service care, Immobilization, Pain management, Reduction

## Abstract

**Einleitung:**

Die präklinische Versorgung verletzter Kinder ist herausfordernd. Ruhigstellung und medikamentöse Schmerztherapie stehen im Vordergrund. Die Erwartungen kinderchirurgischer und unfallchirurgischer Kliniker an Rettungsdienst und Notärzte können jedoch unterschiedlich sein. Die vorliegende Studie wird Gemeinsamkeiten und Unterschiede herausarbeiten.

**Methode:**

Es wurden 186 Mitglieder der Sektion Kindertraumatologie der Deutschen Gesellschaft für Unfallchirurgie im Rahmen einer Umfrage anhand von 10 alltäglichen Fallbeispielen zur Notwendigkeit 9 rettungsdienstlicher Maßnahmen befragt.

**Ergebnisse:**

Es gaben 26 kinderchirurgische und 31 unfallchirurgische Kindertraumatologen ihre Einschätzung ab. Signifikant unterschiedliche Bewertungen gab es zum Einsatz der medikamentösen Schmerztherapie, der Notwendigkeit einer neurologischen Befunderhebung und zu Repositionsmanövern mit Analgosedierung. Hohe Übereinstimmung gab es zur Ruhigstellung der verletzten Körperregion, zur Anlage eines i.v.-Zugangs und zur sterilen Abdeckung offener Frakturen.

**Diskussion:**

Kindertraumatologen mit unfallchirurgischer und mit kinderchirurgischer Ausbildung setzen in ihren Anforderungen an die präklinische Versorgung verletzter Kinder unterschiedliche Prioritäten. Hier sollten gemeinsame, interdisziplinäre Handlungsempfehlungen formuliert werden, um dem oft nicht kindertraumatologisch spezialisierten Rettungsdienstfachpersonal einen breit akzeptierten Leitfaden an die Hand zu geben.

**Graphic abstract:**

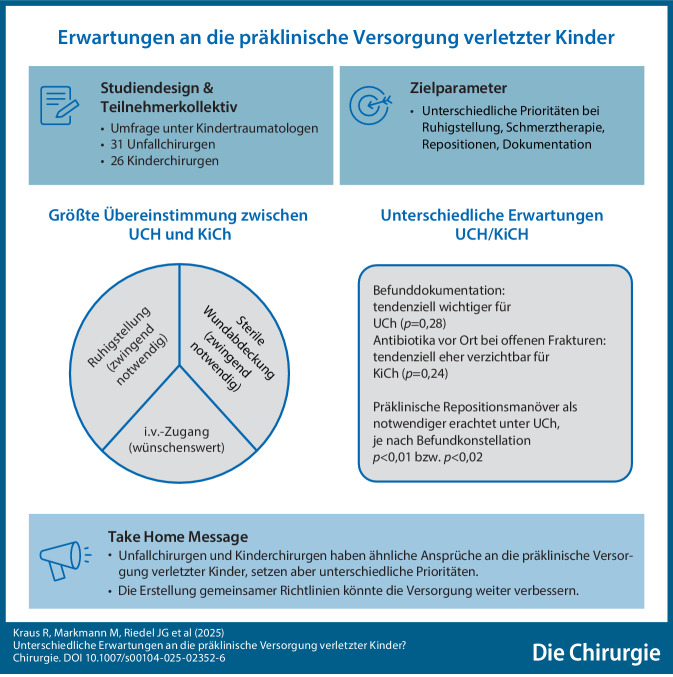

## Abstract (en)

**Introduction:**

The prehospital care of injured children is challenging. Immobilization and pharmacological pain management are in the foreground; however, the expectations of pediatric surgeons and trauma surgeons regarding emergency medical services and emergency physicians can vary. This study aims to identify similarities and differences.

**Methods:**

In this study 186 members of the Pediatric Traumatology Section of the German Society for Trauma Surgery were surveyed on the necessity of 9 emergency medical interventions using 10 routine case studies.

**Results:**

A total of 26 pediatric surgeons and 31 trauma surgeons provided their assessments. There were significant differences in assessment regarding the use of pharmacological pain management, the need for neurological assessment and reduction maneuvers with analgesia. There was high agreement regarding immobilization of the injured body region, the establishment of an intravenous approach and sterile closure of open fractures.

**Discussion:**

Pediatric traumatologists with trauma surgery and pediatric surgery training have different priorities in their requirements for the prehospital care of injured children. Joint interdisciplinary recommendations for action should be formulated to provide widely accepted guidelines for emergency medical personnel, who often do not specialize in pediatric traumatology.

Die Versorgung verletzter Kinder stellt selbst erfahrene Einsatzkräfte vor besondere Herausforderungen: Zum einen müssen sie in einer emotional belastenden Situation auf das Kind und seine Angehörigen eingehen, zum anderen erfordert die Medikamentengabe oft die Anwendung altersadaptierter, ungewohnter Dosierungen. Zudem ist das Legen eines intravenösen Zugangs bei Kindern häufig schwieriger als bei Erwachsenen, sodass auf alternative – und damit ebenfalls weniger routinierte – Applikationswege zurückgegriffen werden muss. Im Vordergrund steht dabei die Schmerzbehandlung, die keine umfassende Diagnostik voraussetzt und bereits durch erste immobilisierende Maßnahmen von Laienhelfern begonnen werden kann. Professionelle Rettungskräfte sehen sich allerdings damit konfrontiert, dass sie bei der innerklinischen Übergabe teils unterschiedliche Erwartungen erfüllen sollen. Dies kann unter anderem damit begründet sein, dass die Behandlung verletzter Kinder in Deutschland gleichermaßen von Unfallchirurgen und Kinderchirurgen durchgeführt wird. Die Sektion Kindertraumatologie der Deutschen Gesellschaft für Unfallchirurgie, gleichzeitig Arbeitsgemeinschaft der Deutschen Gesellschaft für Kinder- und Jugendchirurgie, stellt ein Forum zur Erstellung gemeinsamer Behandlungsrichtlinien dar.

Um die Erwartungen an das präklinische Management von traumatologischen Kindernotfällen durch das Rettungsdienstfachpersonal zu beschreiben, wurde im Rahmen einer Umfrage unter den Mitgliedern der beiden Organisationen anhand von 10 Fallbeispielen aus dem kindertraumatologischen Alltag erfragt, welche präklinischen Maßnahmen von erfahrenen Kindertraumatologen für sinnvoll und notwendig bzw. auch für unsinnig gehalten werden [1]. Die Ruhigstellung verletzter Körperregionen und eine adäquate medikamentöse Schmerztherapie wurden dabei unbedingt befürwortet, während Repositionsmaßnahmen vor Ort ohne Analgosedierung übereinstimmend abgelehnt und auch mit Analgosedierung kritisch betrachtet wurden.

Die gemeinsame Arbeit von Kinderchirurgen und Unfallchirurgen weist immer wieder unterschiedliche Betrachtungsweisen von Diagnostik und Therapie von Verletzungen im Wachstumsalter auf [2]. Als Ursache können verschiedene Perspektiven und Lehrinhalte in der Ausbildung der beiden Berufsgruppen angenommen werden. Ob dies auch in Bezug auf die Erwartungen an eine präklinische Versorgung verletzter Kinder und Jugendlicher zutrifft, soll die vorliegende differenzierte Auswertung der Umfragedaten beleuchten.

## Material und Methoden

### Studiendesign

Es handelt sich um eine Umfragestudie, welche im November 2024 durchgeführt wurde. Hierzu wurden Mitglieder der Sektion Kindertraumatologie (SKT) der Deutschen Gesellschaft für Unfallchirurgie (DGU) mit einem Fragebogen zu 10 alltäglichen Fallbeispielen aus der Kindertraumatologie, wie sie sich vor Ort präsentieren können, befragt.

Auf diesem Wege wurden insgesamt 186 Kolleginnen und Kollegen erreicht, die ein besonderes Interesse an und eine hohe Erfahrung in der Behandlung verletzter Kinder und Jugendlicher haben. Da keine Anonymisierung durchgeführt wurde, konnte aufgrund persönlicher Kenntnis eine hohe Erfahrung der Befragten attestiert werden.

### Umfrage

Der Fragebogen umfasste insgesamt 12 Fragen. Zehn Fragen bezogen sich auf die Fallbeispiele, während 2 Fragen sich auf eine Einschätzung, welcher Grad der Schmerzreduktion prähospital durch geeignete Maßnahmen nach Einschätzung der Teilnehmer erreicht werden sollte, bezog. Röntgenbilder oder andere Zusatzinformationen wurden den Teilnehmern nicht präsentiert. Die Teilnehmenden wurden gebeten, ihre Erwartungen an die präklinische Versorgung anhand von 9 Maßnahmen zu formulieren. Dazu standen die Kategorien zwingend notwendig, wünschenswert, bedarfsweise, verzichtbar und unbedingt vermeiden zur Verfügung (Tab. 1).

**Tab. 1:** *a)* Zehn alltägliche Fallbeispiele (in Stichworten), 9 Maßnahmen zur präklinischen Versorgung (Maßnahme 8 und 9 wurden nur bei den Fallbeispielen mit offenen Verletzungen angeboten), *AS* Analgosedierung. *b)* 5 Kategorien der Notwendigkeit (*in Klammern* die Belegung mit Ziffern zur statistischen Auswertung)

a)	Fallbeispiele	Maßnahmen	b)	Kategorien
1	Ellenbogenverletzung, Puls +	Ruhigstellung	–	Zwingend notwendig (1)
2	Ellenbogenverletzung, Puls −	Analgetikagabe	–	Wünschenswert (2)
3	Oberschenkelfraktur, Kindeswohlgefährdung	intravenöser Zugang	–	Bei Bedarf (3)
4	Distale Unterarmfraktur	Neurologischer Befund	–	Verzichtbar (4)
5	Verdacht auf Halswirbelsäulen-Verletzung	Dokumentation	–	Unbedingt vermeiden (5)
6	Kniebinnentrauma	Reposition ohne AS	–	–
7	Sprunggelenkverletzung, geschlossen, Puls +	Reposition mit AS	–	–
8	Sprunggelenkverletzung, geschlossen, Puls −	Antibiotikagabe	–	–
9	Sprunggelenkverletzung, offen, Puls +	Sterile Wundabdeckung	–	–
10	Sprunggelenkverletzung, offen, Puls −	–	–	–

### Statistik

Die Umfrageergebnisse wurden auf Unterschiede zwischen Teilnehmern mit kinderchirurgischer und mit unfallchirurgischer Provenienz hin untersucht. Die statistische Analyse erfolgte durch einfaktorielle (maßnahmenbezogen, fallunabhängig) oder zweifaktorielle (maßnahmenbezogen und fallabhängig) ANOVA Repeated Measurement-Analyse mit nachfolgendem Tukey HSD-Test.

## Ergebnisse

Insgesamt nahmen 57 Befragte an der Umfrage teil. Bei 200 angeschriebenen Kindertraumatologinnen und Kindertraumatologen ergibt sich eine Rücklaufquote von ca. 28,5 %; 31 Teilnehmer hatten einen unfallchirurgischen, 26 einen kinderchirurgischen Hintergrund. Alle Teilnehmer zeichnen sich durch eine besonders hohe kindertraumatologische Expertise aus.

**Abb. 1:**
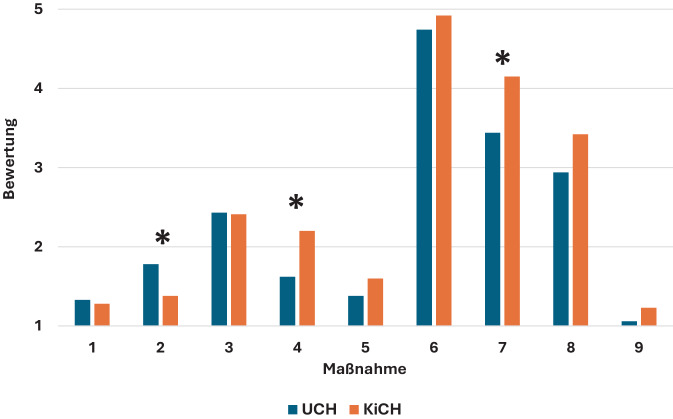
Unterschiedliche, fallunabhängige Einschätzungen zu 9 verschiedenen Maßnahmen (*X‑Achse*). *Y‑Achse* Bewertungen von *1* (zwingend notwendig) bis *5* (unbedingt vermeiden). *Asterisk* Signifikante Differenz (s. auch Tab. 2). *MW* Mittelwert, *SD* Standardabweichung

Bei einer fallunabhängigen Betrachtungsweise unterscheidet sich die Beantwortung der Maßnahmen bei den Fragen 2 (Analgesie, Medikamente Schmerztherapie), 4 (Neurologische Befunderhebung) und 7 (Repositionsversuch mit Analgosedierung) signifikant zwischen den beiden Gruppen (alle *p* < 0,01, Tab. 2; Abb. 1).

**Tab. 2:** Unterschiedliche und ähnliche Bewertungen der Notwendigkeit der präklinischen Maßnahmen 1 bis 9 durch Kinderchirurgen und Unfallchirurgen in der Zusammenfassung aller 10 Beispielfälle

Maßnahme	MW ± SD (KinderCH)		MW ± SD (UnfallCH)		MW ± SD (Gesamt)	*p*-Wert
1	1,28 ± 0,53	Zwingend notwendig	1,33 ± 0,65	Zwingend notwendig	1,3 ± 0,6	1,00
*2*	1,38 ± 0,68	*Zwingend notwendig*	1,78 ± 0,77	*Wünschenswert*	1,6 ± 0,75	< 0,001
3	2,41 ± 1,00	Wünschenswert	2,43 ± 0,88	Wünschenswert	2,42 ± 0,93	1,00
*4*	2,20 ± 1,23	*Wünschenswert*	1,62 ± 0,85	*Wünschenswert*	1,88 ± 1,08	< 0,001
5	1,60 ± 0,89	Wünschenswert	1,38 ± 0,62	Zwingend notwendig	1,48 ± 0,76	0,28
6	4,92 ± 0,40	Unbedingt vermeiden	4,74 ± 0,6	Unbedingt vermeiden	4,82 ± 0,53	0,52
*7*	4,15 ± 1,24	*Verzichtbar*	3,44 ± 1,47	*Bei Bedarf*	3,76 ± 1,41	< 0,001
8	3,42 ± 1,05	Bei Bedarf	2,94 ± 1,04	Bei Bedarf	3,16 ± 1,07	0,24
9	1,23 ± 0,43	Zwingend notwendig	1,06 ± 0,25	Zwingend notwendig	1,14 ± 0,35	1,00

*MW* Mittelwert, *SD* Standardabweichung

Dabei war Kolleginnen und Kollegen mit kinderchirurgischer Ausbildung eine präklinische medikamentöse Schmerztherapie wichtiger als solchen mit unfallchirurgischer Ausbildung. In Bezug auf eine unverzügliche neurologische Befunderhebung verhielt es sich umgekehrt. Ein Repositionsversuch vor Ort unter der Voraussetzung der Anwendung einer Analgosedierung war Unfallchirurgen ebenfalls wichtiger als Kinderchirurgen.

Große Übereinstimmung (*p* = 1,0) bestand dagegen in Bezug auf die Ruhigstellung der verletzten Körperregion (Maßnahme 1) (zwingend notwendig), auf die Anlage eines i.v.-Zugangs (3) (wünschenswert) und die umgehende sterile Abdeckung offener Verletzungen (9) (zwingend notwendig). Bei der Einschätzung, Repositionsmanöver ohne Analgosedierung (6) unbedingt zu vermeiden, waren beide Gruppen mit Abweichungen weitestgehend einig (*p* = 0,52). Tendenziell unterschiedliche, wenn auch nicht statistisch signifikant abweichende Einschätzungen, gab es zur Befunddokumentation (5) (tendenziell für Unfallchirurgen wichtiger, *p* = 0,28) und zur Vor-Ort-Antibiotikatherapie (8) (tendenziell eher verzichtbar bei den Kinderchirurgen, *p* = 0,24; Tab. 2).

Fallbezogene signifikante Unterschiede in den Erwartungen an die präklinische Versorgung ergaben sich v. a. im Falle dislozierter sprunggelenknaher Verletzungen. Bei geschlossenen Frakturen ohne Pulsdefizit (*p* < 0,01) sowie bei offenen Frakturen mit (*p* = 0,02) und ohne Pulsdefizit (*p* < 0,001) hielten die unfallchirurgischen Teilnehmer eine präklinische Reposition signifikant häufiger für notwendig als die kinderchirurgischen Teilnehmer.

Im Falle offener wie geschlossener Sprunggelenkverletzungen mit Pulsdefizit hielten dagegen sowohl Unfall- als auch Kinderchirurgen ein Repositionsmanöver vor Ort für signifikant dringender geboten als bei allen anderen Verletzungsszenarien (*p* < 0,01).

## Diskussion

Diese Studie konnte nachweisen, dass bei den Erwartungen an die rettungsdienstliche Versorgung kinderchirurgischer Notfälle Unterschiede zwischen der unfall- und kinderchirurgischen Betrachtungsweise existieren. Dies ist für das Rettungsdienstfachpersonal eine Herausforderung, da nicht immer ersichtlich ist, welche Fachrichtung den Patienten übernehmen wird. Unterschiedliche Betrachtungsweisen verschiedener Berufsgruppen auf gemeinsam bearbeitete medizinische Problemstellungen sind nicht selten und oft geeignet, bessere Behandlungsabläufe zu etablieren [3–5]. Im deutschsprachigen Raum gelingt dies regelmäßig in Bezug auf kindertraumatologische Fragestellungen im Rahmen der Sektion Kindertraumatologie der Deutschen Gesellschaft für Unfallchirurgie, die gleichzeitig Arbeitsgemeinschaft der Deutschen Gesellschaft für Kinder- und Jugendchirurgie ist. Die Sektion erarbeitet regelmäßig Konsensusreporte zu Fragen der Therapie häufiger Verletzungsmuster im Wachstumsalter [6–8], aber auch zu Fragen der kindgerechten Diagnostik [9].

Eine solche Meinungsfindung in Bezug auf kindgerechte präklinische Maßnahmen gab es allerdings bis dato nicht. Im Rahmen der hier beschriebenen Umfrageuntersuchung konnte gezeigt werden, dass die gesamte Arbeitsgruppe besonderen Wert auf eine unmittelbare Ruhigstellung verletzter Körperregionen als wesentliche Maßnahme der Schmerzlinderung und eine adäquate medikamentöse Schmerztherapie legt [1]. Dennoch gab es bei der Reposition von dislozierten Frakturen insbesondere in der Sprunggelenkregion sowie bei der Gabe von Analgetika signifikante Gruppenunterschiede. Die Ursachen können mit dieser Studie aufgrund von einer fehlenden Umfrageoption zu Begründung nicht definitiv geklärt werden, allerdings lassen sich Erklärungsansätze postulieren. So sind unfallchirurgische Spezialisten auch für die Versorgung von erwachsenen Patienten zuständig und daher regelmäßig mit der Versorgung von komplexen, mehrfragmentären Sprunggelenkfrakturen nach Hochenergietraumata konfrontiert [10]. Solche sind im Kindesalter selten und betreffen dann eher jugendliche Patienten mit ausgereiftem Skelett. Somit könnte es hier seitens der Kinderchirurgie mehr Vorbehalte gegenüber der bei Kindern seltenen Maßnahme der Reposition geben als bei Unfallchirurgen, welche die notfallmäßige Reposition bei erwachsenen Patienten häufiger durchführen müssen.

Andererseits scheint den kinderchirurgischen Experten die Schmerztherapie eher als unverzichtbarer Teil der Versorgung von kindlichen Unterarmfrakturen. Für dieses Ergebnis könnten unterschiedliche Lehrmeinungen und Herangehensweisen im klinischen Alltag ursächlich sein. So ist die Analgetikatherapie sicherlich auch integraler Bestandteil in der Versorgung von erwachsenen Patienten, dennoch erscheinen hier moderate Schmerzen eher zumutbar als bei Kindern. Des Weiteren könnten Kinderchirurgen mit dem Umgang mit Analgetika inklusive Esketamin und Opioiden erfahrener sein, da diese während ihrer Fachweiterbildung zwingend jeweils 6 Monate auf einer Kinderintensivstation und in der pädiatrischen Notaufnahme arbeiten müssen. Unfallchirurgen haben diese Anforderungen nicht und könnten daher in der Nutzen-Risiko-Abwägung eher Bedenken haben, ein Kind zu sedieren oder analgetisch zu behandeln, zumal die Immobilisierung häufig bereits eine effektive Basismaßnahme für die Analgesie darstellt [11].

Zusammenfassend bestehen in den verschiedenen Fachdisziplinen unterschiedliche Schwerpunkte, völlig abweichende Haltungen zwischen den beiden Spezialistengruppen ergaben sich jedoch nicht. Dennoch erscheint es sinnvoll, auch für den Bereich der präklinischen Notfallversorgung verletzter Kinder und Jugendlicher gemeinsame Richtlinien zu formulieren. Ziel sollte es sein, mit der interdisziplinären Abstimmung Handlungsempfehlungen zu erstellen, damit dem Rettungsdienstfachpersonal ein einheitlicher Erwartungshorizont geboten werden kann und somit die Versorgung der anvertrauten Patienten verbessert werden kann. Die interdisziplinäre Zusammenarbeit verbessert nicht nur das Patientenoutcome in der klinischen Praxis, sondern wird bereits seit den 1990er-Jahren auch in der Erarbeitung von Leitlinien und Handlungsempfehlungen gefordert [12, 13]. Aus diesem Grund wird eine Mehrzahl der kindertraumatologischen Leitlinien Konsensus-basiert und interdisziplinär mit Erfolg veröffentlicht [14, 15]. Aus diesem Grund sind Umfragestudien, welche die Diskrepanzen sichtbar werden lassen, für die interdisziplinäre Konsentierung wichtig.

Diese Studie weist einige Limitation auf. Als Erstes ist zu bemerken, dass die Teilnehmer nicht nach der Begründung für die jeweilige Antwort befragt wurden, sodass die Gründe für die identifizierten Unterschiede nicht benannt werden können. Zudem erfolgte die Teilnahme auf freiwilliger Basis, was einen Selektionsbias begünstigen könnte, da engagierte oder besonders interessierte Fachpersonen eher an der Umfrage teilgenommen haben könnten. Eine weitere Limitation liegt in der begrenzten Stichprobengröße, die die Generalisierbarkeit der Ergebnisse auf die Gesamtheit der unfall- und kinderchirurgischen Kollegenschaft limitiert. Dadurch könnte die Repräsentativität der Ergebnisse eingeschränkt sein. Regionale Unterschiede in der präklinischen Versorgungsstruktur oder lokale Behandlungskonzepte wurden nicht erfasst, könnten jedoch einen erheblichen Einfluss auf die individuelle Bewertung bestimmter Maßnahmen haben.

## Schlussfolgerung

Zusammenfassend ließen sich zwischen der Bewertung von Kinder- und Unfallchirurgen einige Unterschiede hinsichtlich der analgetischen Therapie sowie der Reposition dislozierter Frakturen identifizieren, wobei sich eine überwiegend gemeinsame Schnittmenge in den Erwartungen an das Rettungsdienstfachpersonal darstellte. Um diesem einen einheitlichen Erwartungshorizont zu bieten und die Versorgung der Patienten zu optimieren, sollten Handlungsempfehlungen interdisziplinär formuliert werden.

## Data Availability

Die Originaldaten können auf Anfrage zur Verfügung gestellt werden.

## References

[1] Kraus R, Heiß C, Sander M, Schneck E (2025) Prähospitale Maßnahmen in der Kinder- und Jugendtraumatologie – eine Umfragestudie unter Kindertraumatologen, Notfall- und Rettungsmedizin

[2] Kraus R, Röder C, Perler M, Sommerfeldt D, Schneidmüller D, Wessel L, Schnettler R, Linhart WE (2011) Haben Kinderchirurgen und Erwachsenenchirurgen unterschiedliche Therapieansätze bei der Behandlung von Wachstumsfugenlösungen? Zentralbl Chir 136:164–167 20669098 10.1055/s-0030-1247359

[3] Archer KR, Mackenzie EJ, Castillo RC et al (2009) Orthopedic Surgeons and Physical Therapists Differ in Assessment of Need for Physical Therapy after Traumatic Lower-Extremity Injury. Phys Ther 89:1337–1349 19875460 10.2522/ptj.20080200 PMC2794480

[4] Schier F (2009) Kinderchirurgische Eingriffe im Rahmen der Allgemeinchirur gie. Abgrenzung zum Spezialisten. Chirurg Allg 10:69–77

[5] Lee SL, Svdorak RM, Applebaum H (2009) Training general surgery residents in pediatric surgery: educational value vs time and cost. J Pediatr Surg 44:164–168 19159737 10.1016/j.jpedsurg.2008.10.026

[6] Bergmann F, Großer K, Lieber J (2019) (2019) The treatment of elbow dislocations in children and adolescents: Consensus report of the pediatric trauma section of the German Trauma Society (DGU). Unfallchirurg 122(5):369–375 30941439 10.1007/s00113-019-0632-x

[7] Schubert I, Moers K, Fernandez FF, Zwingmann J, Schneidmülller D, Schmittenbecher PP, Strohm PC (2023) Clavicle shaft fractures in childhood and adolescence Consensus report of the Pediatric Traumatology Section of the German Society for Trauma Surgery. Unfallchirurgie 126(3):244–251 36576537 10.1007/s00113-022-01275-9

[8] Rüther H, Strohm PC, Schmittenbecher P, Schneidmüller D, Zwingmann J (2024) Treatment of proximal humeral fractures in childhood and adolescence : Consensus report of the pediatric traumatology section of the German Society for Trauma Surgery. Unfallchirurgie 127(7):547–555 38814464 10.1007/s00113-024-01440-2 PMC11219542

[9] Dresing K, Fernández F, Strohm P, Schmittenbecher P, Kraus R (2021) X‑ray diagnostics of fractures in childhood and adolescence-Consensus report of the scientific working group of the pediatric traumatology section of the German Society for Trauma Surgery (DGU). Unfallchirurg 124(5):427–430 33754172 10.1007/s00113-021-00994-9 PMC8099802

[10] Cao MM, Zhang YW, Hu SY, Rui YF (2022) A systematic review of ankle fracture-dislocations: Recent update and future prospects. Front Surg 9(9):965814. 10.3389/fsurg.2022.965814 36017521 10.3389/fsurg.2022.965814 PMC9398172

[11] Tanabe P, Ferket K, Thomas R, Paice J, Marcantonio R (2002) The effect of standard care, ibuprofen and distraction on pain relief and patient satisfaction in children with musculosceletal trauma. J Emerg Nurs 28:118–125 11960123 10.1067/men.2002.122573

[12] Reeves S, Pelone F, Harrison R, Goldman J, Zwarenstein M (2017) Interprofessional collaboration to improve professional practice and healthcare outcomes. Cochrane Database Syst Rev 6(6):CD72. 10.1002/14651858.CD000072.pub3 28639262 10.1002/14651858.CD000072.pub3 PMC6481564

[13] Grimshaw JM, Russell IT (1993) Effect of clinical guidelines on medical practice: a systematic review of rigorous evaluations. Lancet 342(8883):1317–1322. 10.1016/0140-6736(93)92244-n 7901634 10.1016/0140-6736(93)92244-n

[14] Schmittenbecher PP et al S2K-Leitlinie „Polytraumaversorgung im Kindesalter“ AWMF-Reg.-Nr. 006-120

[15] Ackermann O et al S2e Leitlinie „Fraktursonografie“ AWMF-Reg.-Nr. 085-003

